# The Effect of a Combined Ganciclovir, Methylprednisolone, and Immunoglobulin Regimen on Survival and Functional Outcomes in Patients With Japanese Encephalitis

**DOI:** 10.3389/fneur.2021.711674

**Published:** 2021-11-04

**Authors:** Wang Miao, Junshuang Guo, Shuyu Zhang, Nannan Shen, Xiaoping Shang, Furong Liu, Warren Lu, Jianghai Xu, Junfang Teng

**Affiliations:** ^1^Neuro-Intensive Care Unit, The First Affiliated Hospital of Zhengzhou University, Zhengzhou, China; ^2^Department of Medical Records Management, The First Affiliated Hospital of Zhengzhou University, Zhengzhou, China; ^3^Department of Biology, New York University, New York, NY, United States; ^4^Third Department of Infectious Diseases, Anyang Fifth People's Hospital, Anyang, China

**Keywords:** ganciclovir, methylprednisolone, immunoglobulin, Japanese encephalitis, combined regimen

## Abstract

**Objective:** There is currently no effective treatment for Japanese encephalitis, which has a high rate of morbidity and mortality. This study assessed the effectiveness of a ganciclovir, methylprednisolone, and immunoglobulin combination (TAGMIC) therapy in decreasing cognitive impairment and mortality among patients with Japanese encephalitis.

**Methods:** We retrospectively assessed the clinical data of 31 patients diagnosed with Japanese encephalitis, who were admitted to an intensive care unit. Patients were divided into the TAGMIC and non-TAGMIC group according to their treatment regime. We compared the 60-day, 6-month, and overall mortality and survival curves between groups. We also compared Barthel Index scores, Montreal Cognitive Assessment (MoCA) scores, and diffusion tensor imaging (DTI) results.

**Results:** There was no significant difference in the 30-day mortality rate or Kaplan–Meier survival curve between groups. The 60-day, 6-month, and overall mortality rates in the TAGMIC group were significantly reduced (*P* = 0.043, *P* = 0.018, and *P* = 0.018, respectively) compared with the non-TAGMIC group (0, 0, 0 vs. 31.25, 37.5, 37.5%, respectively). The 60-day, 6-month, and overall Kaplan–Meier survival curves were significantly different between groups (*P* = 0.020, *P* = 0.009, *P* = 0.009, respectively). There was no significant difference in the Barthel Index scores of surviving patients. Among the five patients who underwent MoCA and DTI, four had a score of 0/5 for delayed recall (no cue), while the remaining patient had a score of 2/5. All five patients were able to achieve a score of 5/5 with classification and multiple-choice prompts, and had sparse or broken corpus callosum (or other) fibre bundles.

**Conclusion:** TAGMIC treatment can reduce mortality due to severe Japanese encephalitis. The memory loss of surviving patients is mainly due to a disorder of the memory retrieval process, which may be related to the breakage of related fibre bundles.

## Introduction

Japanese encephalitis (JE) is an endemic mosquito-borne infection caused by the Japanese encephalitis virus (JEV) (1). JEV is classified as a flavivirus, and causes peripheral neuropathy and brain damage (2, 3). Although vaccines can prevent JEV infection, approximately 68,000 cases are reported annually. According to the World Health Organization (4), treatment is mainly supportive and targeted toward symptom relief, as there are no effective antiviral drugs. The outcome of JE is poor; the mortality rate may be up to 30%, and permanent neurological or psychiatric sequelae may occur in 30–50% of surviving patients (5).

The JEV is a positive-sense single-stranded ribonucleic acid (RNA) virus. Anti-RNA viral drugs generally have poor therapeutic effects (6, 7), and it is necessary to develop new or screen existing antiviral drugs to improve clinical efficacy (8). In addition to direct tissue damage, JEV can also cause severe inflammation and a heightened immune response (9); therefore, drugs targeting only a single pathogenesis mechanism are likely to result in treatment failure. There is an urgent need for an effective treatment regimen to reduce mortality and disability in patients with JE.

While cognitive impairment is a serious problem in survivors of JE, a standard assessment measure is lacking. The Montreal Cognitive Assessment (MoCA) has been shown to be effective for the evaluation of cognitive impairment after stroke and dementia (10). Diffusion tensor imaging (DTI) is a non-invasive method for the assessment of white matter fibre bundles, and plays an increasingly important role in the study of brain development and cognitive function (11). Nevertheless, no prior studies have utilized MoCA and DTI to evaluate cognitive impairment in survivors of JE. Confirmation of the utility of the MoCA and DTI in this patient group may facilitate the development of more effective therapies for cognitive impairment.

In recent years, we have used a ganciclovir, methylprednisolone, and immunoglobulin combination (TAGMIC) therapy in the early stages of treatment for JE. Preliminary evidence suggests that this combination therapy may reduce mortality and improve long-term prognosis. The objective of this study was to evaluate the effectiveness of TAGMIC in reducing mortality in patients with JE, as well as its ability to improve both long-term physical function and cognitive impairment, as reflected by MoCA and DTI.

## Materials and Methods

### Patient Selection

**Figure 1:**
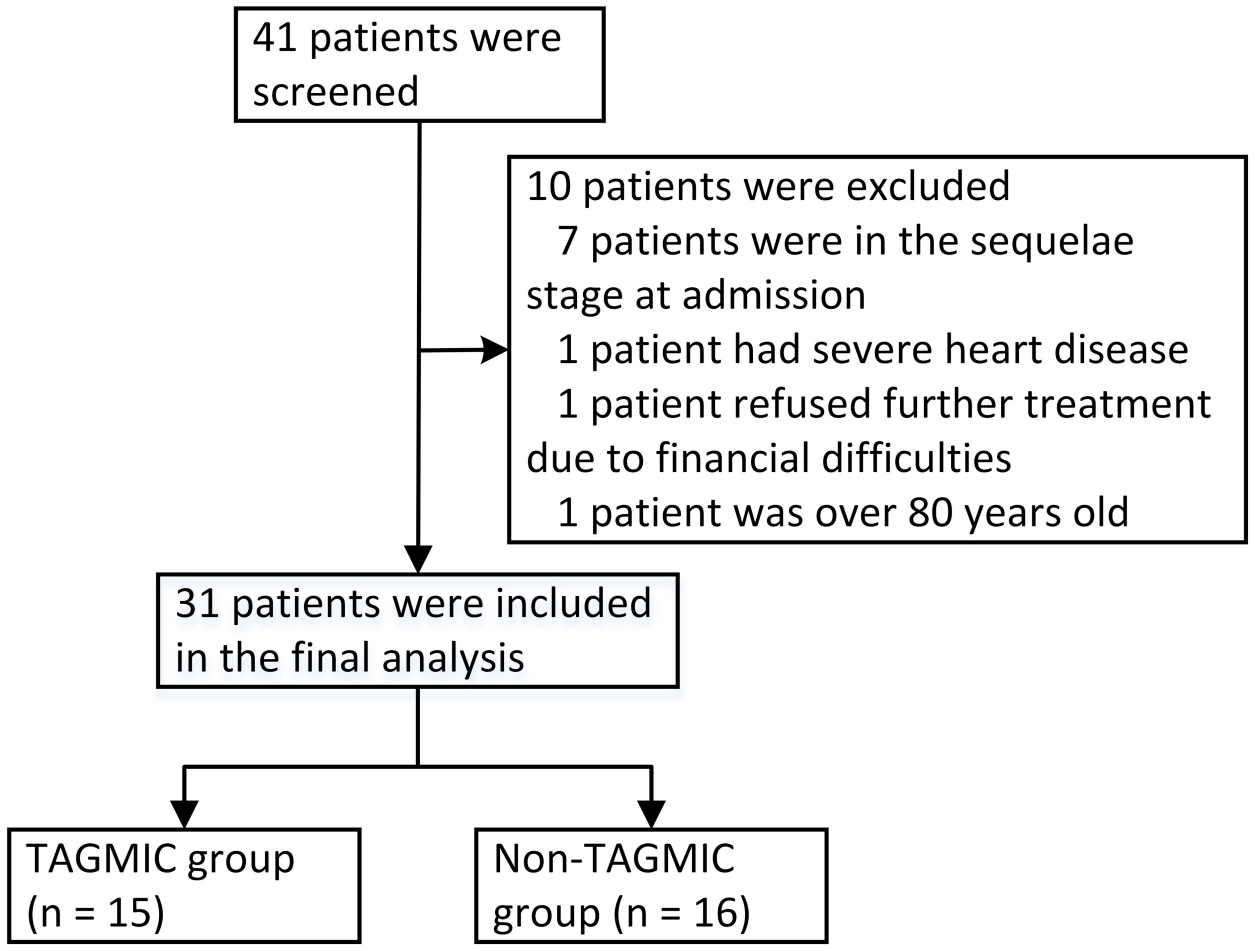
Flow chart of patient inclusion, exclusion, and grouping. TAGMIC: ganciclovir, methylprednisolone, and immunoglobulin combination.

We retrospectively assessed the clinical data of 41 patients diagnosed with JE, who were admitted to the intensive care unit of the First Affiliated Hospital of Zhengzhou University, from August 2013 to August 2019. These patients have tested blood and cerebrospinal fluid for anti-JEV-IgM, and the results are positive (Completed by the Henan Provincial Center for Disease Control and Prevention).Patients meeting the diagnostic criteria for JE were included (12). Exclusion criteria comprised patients (1) in the sequelae period of JE at admission; (2) with other central nervous system infectious diseases or serious comorbidities (e.g., malignant tumour, severe heart disease); (3) who abandoned treatment due to economic reasons; and (4) over 80 years of age. A total of ten patients were excluded with these criteria, resulting in a final sample size of 31 patients (Figure 1).

Patients were divided into two groups, according to whether they had received a timely and adequate course of treatment with TAGMIC: TAGMIC (*n* = 15) and non-TAGMIC group (*n* = 16). The mortality rates and survival curves were compared between the two groups at 60 days, 6 months, and overall follow-up. Barthel Index scores of surviving patients were also compared between groups.

TAGMIC therapy comprised the following: (T) treatment with ganciclovir and methylprednisolone within 1 week of JE onset, and the initiation of immunoglobulin therapy within 2 weeks of JE onset; (A) application of ganciclovir and methylprednisolone for at least 5–7 days in combination; (G) ganciclovir (5 mg/kg) administered via intravenous drip, q12h, for 2–3 weeks; (M) Methylprednisolone: 2 mg/kg/d, intravenous drip, the total dose is divided into two applications (q12h) for 5 days; then 1 mg/kg/d intravenous drip, qd, lasting 3 days; then 0.5 mg/kg/ d, intravenous drip, qd, stop the drug after 3 days; (I) Intravenous immunoglobulin (IVIG) provided at 0.4 g/kg/d for 5 days, followed by 5–10 g/d for 1 week or longer; and (C) combined administration of the three drugs. Both the TAGMIC and non-TAGMIC groups received symptomatic and supportive treatment in the intensive care unit. According to the patient's condition, supportive treatments such as blood pressure control, nutritional support, antibiotics treatment, respiratory support, sedation and analgesia are given.

### Data Collection

Clinical patient data were collected from the medical record system, and telephone or face-to-face follow-up was conducted to document survival time, survival outcomes, and sequelae. We recorded the Barthel Index score for surviving patients at 1 year after JE onset. A total of 25 patients agreed to undergo head magnetic resonance imaging (MRI) during hospitalisation. Of the five patients who underwent in-hospital DTI, three returned to our hospital for DTI review. During the 3-year follow-up, five patients agreed to receive the Chinese version of the MoCA for cognitive function evaluation.

### Statistical Analysis

Normal distribution tests and variance homogeneity tests were conducted for measurement data. Normal distribution, skewed distribution, and count data are expressed as mean ± standard deviation, median (lower quartile, upper quartile), and frequency (constituent ratio), respectively. Between-group comparisons were made using the *t*-test, χ^2^-test, and Mann–Whitney *U*-test. Kaplan–Meier survival curves of the two groups were analysed with the log-rank test. The level of statistical significance was set at *P* < 0.05. All analyses were performed with SPSS software (version 25.0) and R software (version 4.0.2).

## Results

### Comparison of Baseline Data Between the TAGMIC and Non-TAGMIC Groups

**Figure 2:**
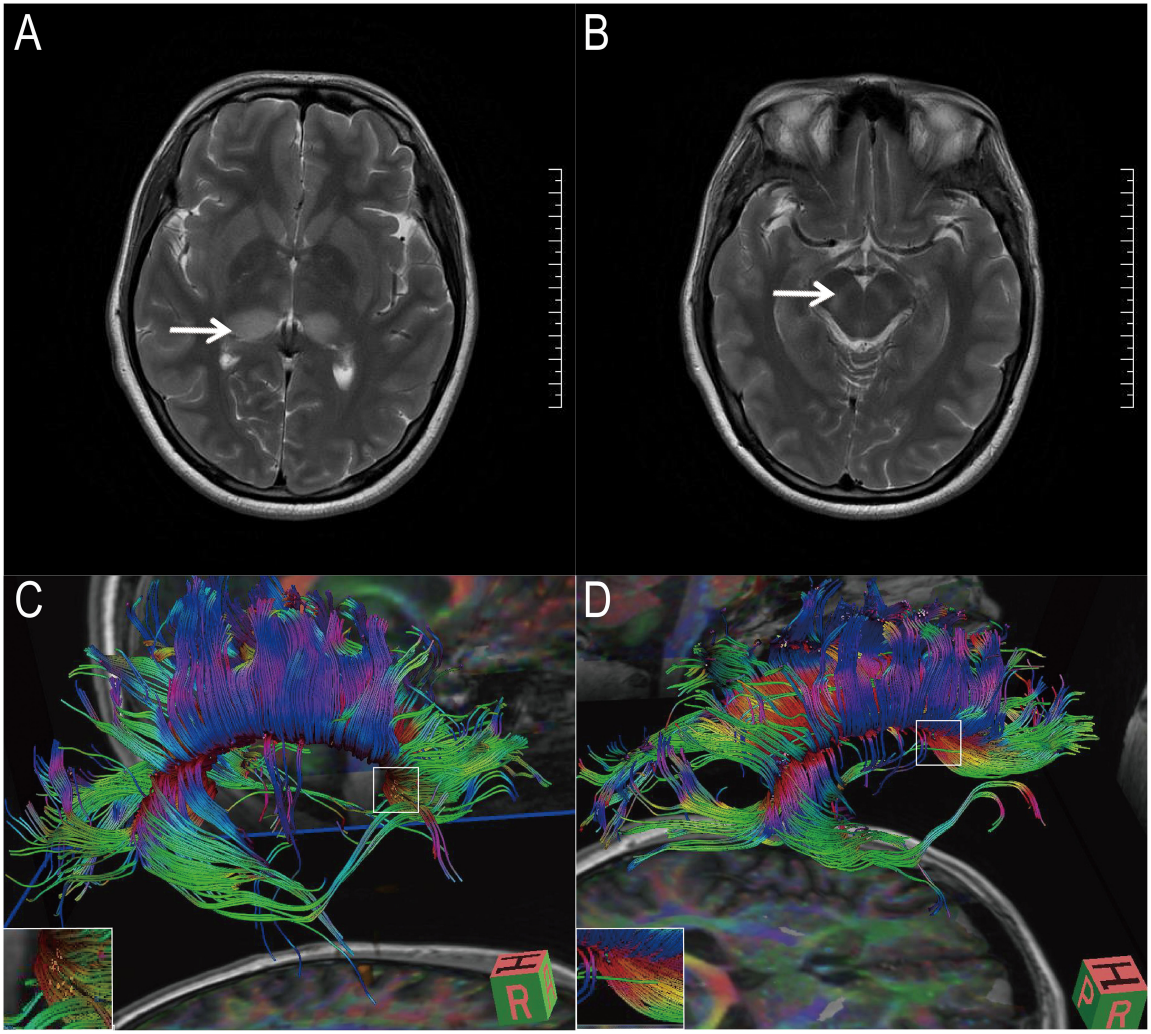
Magnetic resonance image and diffusion tensor imaging image of 1 patient. **(A,B)** Magnetic resonance images showed that the patient's thalamus (white arrow) and basal ganglia (white arrow) were lesions. **(C,D)** Diffusion tensor imaging showed that the patient's corpus callosum tract was partially broken. Three months later, the patient's corpus callosum rupture improved.

There were no significant differences in clinical or laboratory parameters between the TAGMIC and non-TAGMIC groups at baseline (*P* > 0.05; Table 1). A total of 25 patients underwent head MRI during hospitalisation; JE was observed to have mainly affected the thalamus (76%) and basal ganglia (56%) (Figures 2A,B). Five patients agreed to undergo in-hospital DTI, which revealed that many of the fibre tracts in the brain were broken; affected regions included the corticospinal tract, corpus callosum tract, arcuate bundle, inferior frontal occipital tract, cingulate bundle, and uncinate bundle. Three patients returned for review at 3 or 6 months. DTI showed that the fibre bundle continuity was improved (Figures 2C,D and Table 2).

**Table 1:** Comparison of baseline data between the TAGMIC and non-TAGMIC groups.

	**TAGMIC** **(*n* = 15)**	**Non-TAGMIC** **(*n* = 16)**	***P*-value**
Male (%)	9 (60.0)	7 (43.8)	0.479
Age	48.067 ± 18.510	53.000 ± 16.017	0.433
Temperature (°C)	39.4 ± 0.6	39.6 ± 0.8	0.495
GCS at admission	7 (6–12)	7 (3–12)	0.288
Days from symptom onset to admission	4 (3–5)	5 (4–9)	0.111
ICU duration (days)	21.267 ± 9.223	18.063 ± 9.546	0.350
Days from symptom onset to ICU admission	4 (3–5)	5 (4–10)	0.058
Headache (%)	8 (53.3)	7 (43.8)	0.724
Vomit (%)	6 (40.0)	8 (50.0)	0.722
Respiratory failure (%)	10 (66.7)	10 (62.5)	1.000
Pathological reflex (%)	2 (13.3)	3 (18.8)	1.000
Meningeal irritation (%)	12 (80.0)	8 (50.0)	0.135
Seizures (%)	4 (26.7)	4 (25.0)	1.000
Increased muscle tone (%)	5 (33.3)	6 (37.5)	1.000
Decreased muscle tone (%)	0 (0.0)	3 (18.8)	0.226
Diabetes (%)	1 (6.7)	1 (6.3)	1.000
Hypertension (%)	7 (46.7)	3 (18.8)	0.135
**Blood exams (*****n*** **= 31)**	**TAGMIC** **(*****n*** **= 15)**	**Non-TAGMIC** **(*****n*** **= 16)**	***P*** **-value**
Leukocyte (10∧9/L)	10.800 ± 3.601	12.688 ± 5.535	0.273
Neutrophil (10∧9/L)	9.100 (6.710–11.200)	9.550 (7.003–14.100)	0.429
Lymphocyte (10∧9/L)	0.800 (0.600–1.500)	0.930 (0.480–1.000)	0.566
Monocyte (10∧9/L)	0.890 (0.210–1.140)	0.695 (0.480–1.003)	0.635
ALT (U/L)	23 (20–41)	31 (20–51)	0.527
AST (U/L)	34 (20–54)	38 (26–60)	0.566
Total protein (g/L)	71.180 ± 9.206	68.075 ± 7.485	0.310
Albumin (g/L)	39.560 ± 5.470	36.775 ± 4.962	0.148
Globulin (g/L)	29.9 (24.6–40.2)	29.7 (27.8–32.9)	0.767
PT (s)	11.4 (11.2–12.5)	11.3 (10.5–12.0)	0.363
APTT (s)	29.6 (27.4–31.2)	29.6 (27.0–31.4)	0.828
CRP (mg/L)	38.38 (14.06–66.36)	24.55 (7.30–41.16)	0.277
**Imaging lesions (*****n*** **= 25)**	**TAGMIC** **(*****n*** **= 14)**	**Non-TAGMIC** **(*****n*** **= 11)**	***P*** **-value**
Thalamus (%)	13 (92.9)	6 (54.5)	0.056
Basal ganglion (%)	8 (57.1)	6 (54.5)	1.000
Hippocampus (%)	4 (28.6)	1 (9.1)	0.341
Brain stem (%)	7 (50.0)	4 (36.4)	0.689
Cerebral cortex (%)	6 (42.9)	6 (54.5)	0.695
**CSF analysis (*****n*** **= 30)**	**TAGMIC** **(*****n*** **= 15)**	**Non-TAGMIC** **(*****n*** **= 15)**	***P*** **-value**
Pressure (mmH_2_O)	185 (150–250)	200 (165–270)	0.740
Leukocyte (10∧6/L)	42 (24–140)	24 (10–96)	0.191
Lymphocyte (%)	74 (56–82)	74 (66–78)	0.852
Monocyte (%)	15.13 ± 10.32	19.07 ± 7.69	0.246
Protein (mg/dL)	66.3 (51.2–98.5)	84.8 (67.1–123.6)	0.152
Glucose >50 (mg/dL)	14 (93.3)	14 (93.3)	1.000

*The blood and cerebrospinal fluid test results of the two groups were the initial test results during hospitalization.TAGMIC, ganciclovir, methylprednisolone, and immunoglobulin combination; GCS, Glasgow Coma Score; ICU, Intensive care unit; ALT, alanine aminotransferase; AST, aspartate amino transferase; PT, prothrombin time; APTT, activated partial thromboplastin time; CRP, C-creative protein; CSF: cerebrospinal fluid*.

**Table 2:** Diffusion tensor imaging results of five patients.

**No/age/sex**	**Corticospinal tract**	**Corpus callosum tract**	**Arcuate fasciculus**	**Inferior frontal occipital tract**	**Cingulate bundle**	**Uncinate bundle**
1/58/F	Yes	Yes	Yes	No	Yes	No
2/47/M	Yes	Yes	No	Yes	Yes	Yes
	Yes	Yes	No	No	Yes	Yes
3/28/M	Yes	Yes	No	Yes	Yes	Yes
	Yes	Yes	No	Yes	No	No
4/67/M	Yes	Yes	Yes	Yes	No	Yes
5/27/F	Yes	Yes	No	Yes	Yes	No
	Yes	No	No	Yes	Yes	No

*Patient 2 underwent DTI reexamination half a year after disease onset. Patients 3 and 5 underwent DTI reexamination 3 months after disease onset. The second row of data for a patient includes results obtained upon DTI reexamination. Yes, There are sparse or broken fiber bundles; No, No fiber bundles are sparse or broken. DTI, Diffusion tensor imaging; M, male; F, female*.

### Comparison of Mortality and Survival Curves Between the TAGMIC and Non-TAGMIC Groups

**Figure 3:**
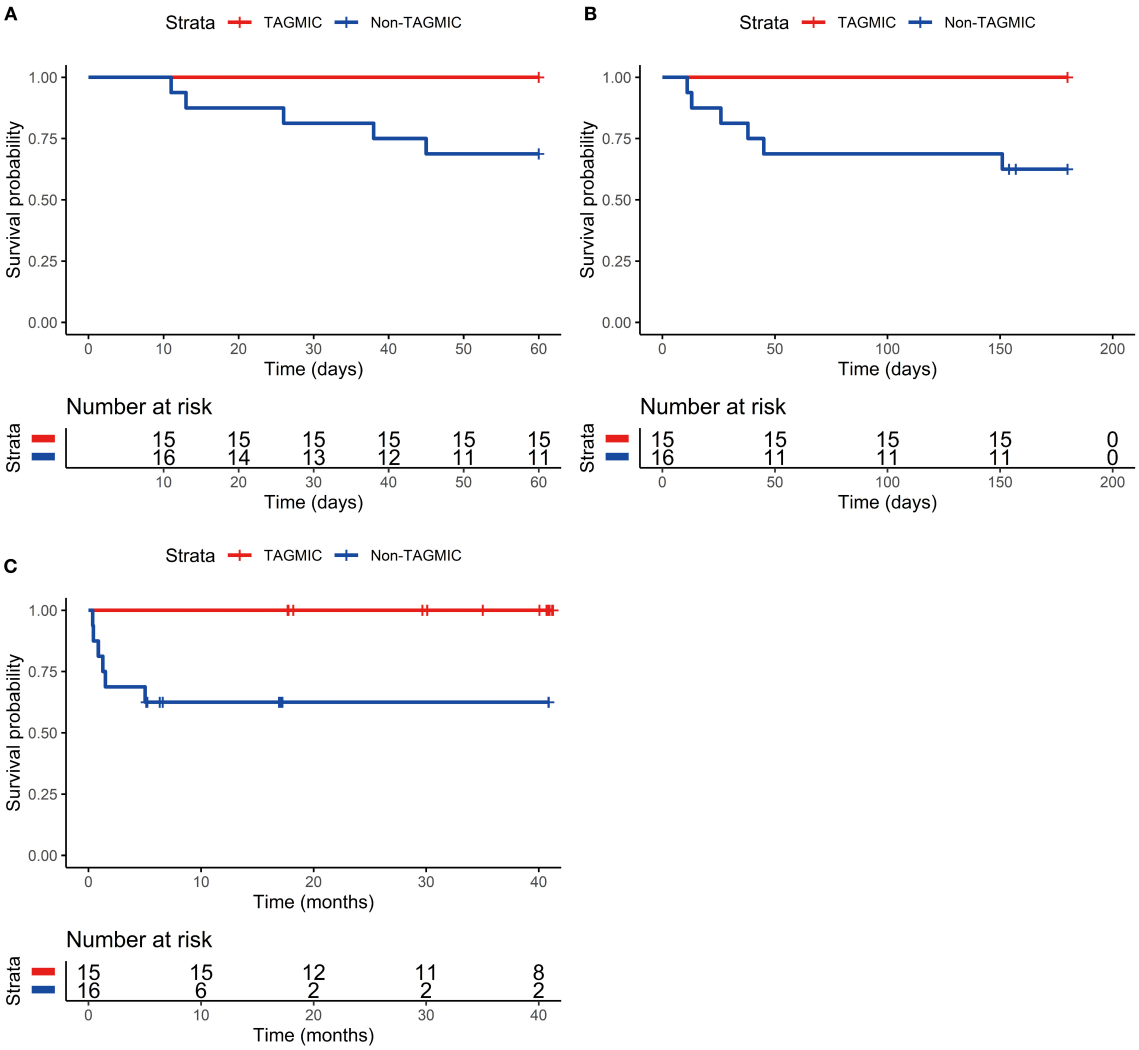
Comparison of 60-day, 6-month, and overall survival curves between TAGMIC group and Non-TAGMIC group. **(A)** Comparison of 60-day survival curves between TAGMIC group and Non-TAGMIC group. **(B)** Comparison of 6-month survival curves between TAGMIC group and Non-TAGMIC group. **(C)** Comparison of overall survival curves between TAGMIC group and Non-TAGMIC group. **(C)** Shows that due to the different dates of onset of patients, as of the end time of our follow-up (January 2020), the follow-up period of patients ranges from several weeks to several years. At the 10-month follow-up, 6 patients in the Non-TAGMIC group died (the part of the curve descending), and 4 patients were shorter than 10 months by the end of January 2020 (4 short vertical lines, 2 of which are almost overlapped due to the close distance.) Therefore, 6(16-6-4) patients over 10 months, need to continue to extend the display on the curve. There were no deaths in the TAGMIC group and no decline in the survival curve. For example, in the TAGMIC group, there are 3 patients with a follow-up period of fewer than 20 months (3 short vertical lines), and 12(15-3) patients with a follow-up period of more than 20 months, which need to be extended on the curve. TAGMIC: ganciclovir, methylprednisolone, and immunoglobulin combination.

During the course of treatment, we found no adverse reactions such as allergic reactions and liver damage through clinical observations and biochemical examinations. While mortality due to JE was not observed in the TAGMIC group, two and three patients in the non-TAGMIC group died within 1 and 2 months of JE onset, respectively; a single case of mortality was documented within 5 months of JE onset. The 30-day mortality rate in the TAGMIC group was lower than that in the non-TAGMIC group (0 vs. 12.5%); however, this did not reach statistical significance (*P* = 0.226). There was also no significant difference in the 30-day Kaplan–Meier survival curve between the two groups (*P* = 0.083). The 60-day mortality rate in the TAGMIC group was significantly lower than that in the non-TAGMIC group (0 vs. 31.25%, *P* = 0.043), and the difference in the 60-day Kaplan–Meier survival curve was also significant (*P* = 0.020; Figure 3A). The 6-month mortality rate was also significantly lower (0 vs. 37.5%, *P* = 0.018) in the TAGMIC group, with a corresponding significant difference between Kaplan–Meier survival curves (*P* = 0.009; Figure 3B). The TAGMIC group exhibited a significantly lower total mortality rate (0 vs. 37.5%, *P* = 0.018); the overall Kaplan–Meier survival curve was also significantly different between groups (*P* = 0.009; Figure 3C).

### Comparison of the Barthel Index Score Between the TAGMIC and Non-TAGMIC Groups

There were a total of 15 and 10 surviving patients in the TAGMIC and non-TAGMIC groups at the 1-year follow-up, respectively. One patient in the TAGMIC group had a Barthel Index score of 10, while the rest had a score of 100. In the non-TAGMIC group, one patient had a score of 55, and the remainder had a score of 100. There was no significant difference in the Barthel Index score between groups (*P* = 0.814).

### Evaluation of Impaired Cognition

All five patients who agreed to undergo the MoCA complained of memory loss. The MoCA test results mainly indicated a delayed recall disorder. Four patients had a score of 0/5 for delayed recall (no cue), and one patient had a score of 2/5. All patients were able to recall the remaining words with the provision of the classification prompt and multiple-choice prompt. Visual space and executive ability were slightly impaired in four cases, while the remaining patient exhibited significant impairments in visual space and executive ability, naming function, and attention (Table 3).

**Table 3:** Montreal Cognitive Assessment scores of five patients.

**No/age/sex**	**Education (years)**	**Visuospatial/ executive**	**Naming**	**Attention**	**Language**	**Abstraction**	**Delayed recall**			**Orientation**	**Score after correction**
							**No cue**	**Category cue**	**Multiple choice cue**		
1/61/F	12	2	1	2	3	1	0	3	2	6	16
2/50/M	9	4	3	6	2	2	0	3	2	6	24
3/31/M	12	4	3	6	3	2	2	3	0	6	27
4/70/M	6	5	3	6	3	2	0	0	4	4	24
5/30/F	6	4	3	6	3	2	0	1	4	6	25

*One point was allocated for each word recalled freely without any cues. No points were allocated for words recalled with a category cue or multiple-choice cue. M, male; F, female*.

## Discussion

Currently, treatment for JE is mainly supportive and targeted toward symptom relief, as there are no effective antiviral drugs. Therefore, surviving patients often have serious neurological sequelae and cognitive impairment (4, 5). The results of this study suggested that the TAGMIC treatment regimen significantly reduced the 60-day, 6-month, and overall mortality of patients with severe JE, thereby improving the survival rate. Furthermore, the long-term memory impairment in some of the patients was mainly attributed to a disorder of the memory retrieval process, as opposed to the memory storage process. This may have been related to the damage and breakage of contact fibres in the brain.

**Figure 4:**
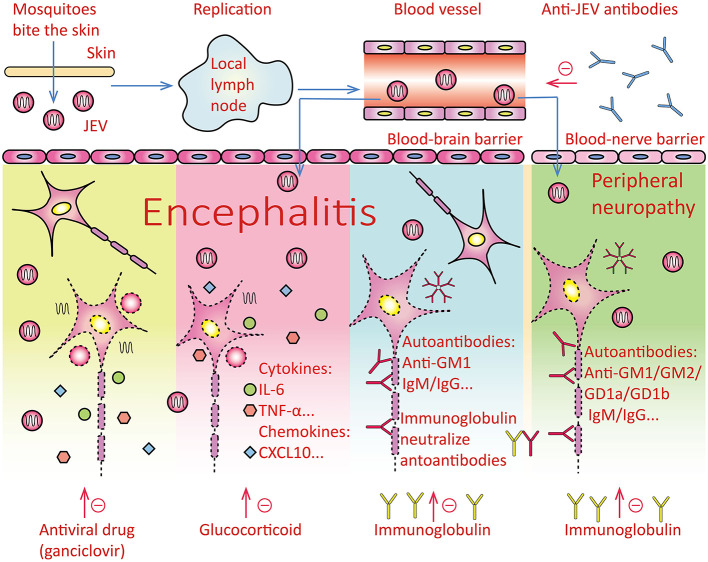
Overview of pathogenesis and therapeutic drugs of Japanese encephalitis. Pathogenesis of disease: After the mosquito bites the human skin, it enters the lymph nodes and replicates in the lymph nodes, and then enters the blood system to form viremia, which stimulates the body to produce protective antibodies against the Japanese encephalitis virus. The virus mainly causes the following three aspects of damage, and the three drugs act on the following three aspects respectively. 1. The virus penetrates the blood-brain barrier and enters the central nervous system. The virus directly damages neurons. The antiviral drug ganciclovir may inhibit the replication of Japanese encephalitis virus and reduce the damage of the virus to neurons. Antiviral drugs may act on this pathogenesis. 2. After the virus enters the human body, the body produces IL-6, TNF-α, TLRs, and other cytokines and chemokines, causing inflammation of the whole body and brain parenchyma. Glucocorticoids fight inflammation. 3. The composition of the virus may be similar to that of the central nervous tissue, which stimulates the body's immune system to produce autoantibodies against its nervous tissue components, such as Anti-GM1 IgM/IgG and so on. The virus can also stimulate the body to produce autoantibodies against peripheral nerve tissue components, such as Anti-GM1 IgM/IgG, Anti-GM2 IgM/IgG, Anti-GD1a IgM/IgG, Anti-GD1b IgM/IgG, etc. Intravenous immunoglobulin mainly contains IgG monomer, which is used to neutralize autoantibodies such as IgM/IgG. JEV, Japanese encephalitis virus; IL-6, interleukin-6; TNF-α, tumour necrosis factors-α; TLRs, Toll-like receptors.

JEV causes physiological damage to host tissues in three ways (Figure 4). First, the virus directly enters the central nervous system and damages neurons, especially those in the thalamus and basal ganglia. Prior studies have reported that JEV destroys neurons through clathrin-independent endocytosis and apoptosis (13–15). In the present study, we also observed damage in the substantia nigra and hippocampus. Second, JEV causes severe inflammation and cytokine storms in the central nervous system (16), and destroys the blood-brain barrier (17). This may be one of the reasons for the rapid emergence of severe symptoms (e.g., impaired consciousness), and indicates the importance of blocking the inflammatory storm response in the early stages of JE. Third, the continued development of the infection may cause a severe humoral immune response in the central and peripheral nervous systems, which further exacerbates clinical symptoms and cognitive impairment. Previous studies have reported increases in immunoglobulins M and G in the cerebrospinal fluid, as well as circulating B lymphocytes and plasma cells (6, 18, 19). Therefore, optimal treatment requires consideration of all three pathogenic mechanisms of JE, and should comprise not only an early anti-viral effect, but also counteractive measures for the subsequent inflammatory reaction, cytokine storm, and humoral immune response. The TAGMIC regimen combines the use of ganciclovir (an antiviral agent) with methylprednisolone to reduce the inflammatory reaction and cytokine storm within 1 week; this is complemented by the administration of immunoglobulin to significantly reduce clinical symptoms within 2 weeks, and the severity of neurological damage.

Single-stranded RNA virus replication lacks a proofreading mechanism, and is therefore more prone to mutations. Therefore, a combination of drugs is often required to treat infections caused by this virus group, which includes hepatitis C virus, severe acute respiratory syndrome (SARS)-associated coronavirus, and SARS coronavirus 2 (SARS-CoV-2). Each individual drug in the regimen acts on different pathogenic aspects of the infection, as well as synergistically. JE pathogenesis has been shown to be extremely complex and diverse, thus necessitating the use of combined drug therapy (6). Therefore, in the process of treating JE at our institution, we have adopted the use of a combination of antiviral drugs, glucocorticoids, and immunoglobulins to target different aspects of its pathogenesis.

There are currently no effective antiviral drugs for patients with JE. A prior study reported that oral ribavirin was unable to reduce the mortality rate in patients with JE (20). In contrast, more successful results have been observed for ganciclovir, which is used as an anti-deoxyribonucleic acid viral agent. An *in vitro* study reported that ganciclovir is a prodrug of nucleotide analogues, and is delivered into cells and converted into ganciclovir triphosphate. RNA-dependent RNA polymerase subsequently incorporates ganciclovir triphosphate into the polymerase reaction, which is immediately terminated, thereby inhibiting the replication of SARS-CoV-2 (8). Similarly to SARS-CoV-2, JEV is a single-stranded positive-stranded RNA virus, which requires a RNA-dependent RNA polymerase for replication (21). The results of the present study support the anti-JEV effectiveness of ganciclovir.

The results of the present study suggest that methylprednisolone is able to rapidly reduce the inflammatory reaction and cytokine storm induced by JEV. Following entry into the central nervous system, JEV stimulates the release of a large number of inflammatory cytokines and chemokines through the degranulation of mast cells, and activation of lymphocytes and macrophages. This not only damages the blood-brain barrier, but also induces a large number of inflammatory cells to infiltrate around the blood vessels (17, 22). Therefore, the microglia are activated, and the microglia phagocytize neurons or release toxic substances to cause the death of neurons (23–25), this further exacerbates damage to the central nervous system. Indeed, prior studies have shown that the prognosis of patients with JE is poorer with higher levels of inflammatory mediators (TNF-α, IL-6) in the central nervous system (9, 26). Glucocorticoids have been demonstrated to down-regulate the release of cytokines (TNF-α, IL-6) and inflammatory mediators (27), thus inhibiting further damage to the central nervous system and facilitating the recovery of consciousness (28). A previous study also reported that dexamethasone can prevent damage to the blood-brain barrier and protect the central nervous system (29). The early and continuous administration of glucocorticoids has also been shown to effectively reduce the damage caused by inflammatory storms in patients with SARS (30, 31). The patients in our study were all in the severe stage, with severe central nervous system disease, and most had respiratory failure, acute respiratory distress syndrome, or other respiratory diseases. Therefore, glucocorticoids should be administered early to reduce the damage caused by the inflammatory storm.

Immunopathology is considered to be the main pathogenic mechanism of JE (9, 32). Humans usually have a strong physiological immune response to JEV, which may be divided into two different components. The first component is the viral antigen; B cells and plasma cells increase significantly in patients with severe virus infection, reflecting the activation of humoral immunity (16). Immunoglobulins M and G appear after the onset of JEV infection to fight the virus. However, if the virus load is too large, the deposition of viral antigens and the corresponding antibodies in the nerve tissues and blood vessel walls activates the complement system, which results in immune damage and brain tissue necrosis. The second component is the production of antibodies against the patient's nerve tissue. A prior study (19) reported that 30% of patients with JEV infection develop antibodies to their gangliosides (GM1, GM2, GD1a, and GD1b), resulting in Guillain-barre syndrome. Thus, the continued progression of JEV into the later stages may result in the generation of auto-antibodies, which damage peripheral nerves and increase the risk of disability.

IVIG is composed mostly of monomeric immunoglobulin G (95%), with a small amount of aggregated and dimeric forms. IVIG can provide anti-idiotypic antibodies (18) that bind to and neutralise pathogenic antibodies, thereby preventing them from interacting with antigens and reducing the formation and deposition of antigen-antibody complexes in the nerve tissues (33). These antibodies may neutralise and block not only viral antigens, but also secondary neural tissue antigens in the later stages of JE, thus reducing further damage to the nervous system. IVIG can also inhibit complement; prevent the formation of membrane soluble attack complexes; provide anti-inflammatory effects; induce anti-inflammatory cytokines; reduce complement-mediated damage; reduce immune complex-mediated inflammation; and regulate T cell and B cell activation and differentiation (34). The ability of IVIG to neutralise antibodies in JEV-positive patients has been previously demonstrated in a randomised double-blind placebo-controlled trial (35). Another study (19) reported that the administration of IVIG within 2 weeks of symptom onset could effectively treat Guillain-barre syndrome associated with JEV; after 1 week of treatment, 54% of patients experienced significant improvements in clinical signs and symptoms. In our study, we administered a high dose for 5 days, and continued to provide IVIG at 5–10 g/d for 1 week; this may have led to the complete neutralisation of the autoantibodies and a large reduction in damage to the central nervous system.

The TAGMIC regimen addresses not only the different aspects of JE pathology, but also the timeliness and completeness of treatment. While therapy should be initiated early, its duration should also be long enough to cover the entire process of the severe pathological changes. The use of this approach in the present study greatly facilitated the long-term reduction of mortality in patients with severe JE. While prior studies have investigated short-term outcomes and their associated factors among JE survivors (36, 37), it must be acknowledged that some neurological and psychiatric sequelae do not manifest in the short-term, prior to hospital discharge. Therefore, long-term follow-up is required to accurately and comprehensively assess outcomes in this patient group. In the present study, we reviewed patients for approximately 3 years, in order to document the long-term motor, neurological, and cognitive sequelae of JE.

As mentioned in the results section, the two groups of patients had high Barthel Index scores. This reflects that the patient has better functions in daily exercise such as dressing, going up and down stairs, etc. While we did not document residual motor impairments that were severe enough to affect activities of daily living, cognitive impairments (e.g., memory loss) were observed in some patients. In the majority of cases, these impairments gradually resolved through active rehabilitation and training. However, during the 3-year follow-up, some patients still had complaints of memory loss, especially with regard to recent events. In order to further elucidate the cause of this memory loss, we performed additional investigations in five patients. We found that in addition to a slight decrease in visual space and executive ability, these patients also had a significant decrease in delayed recall of recent events; nevertheless, they were able to complete the memory retrieval process with the provision of classification and selection prompts. This phenomenon is similar to the amnesia reported in mice with early Alzheimer's disease, in that the memory retrieval process is impaired, while the memory storage process remains intact (38). As indicated by the DTI results, this may be related to the breakage of multiple connecting fibres in the corpus callosum tract and arcuate tract in the brain. As memory function requires unilateral and bilateral information transmission through the corpus callosum (39), damage to the integrity of this structure may impair memory. The improvement of the patients' remaining symptoms also suggests that the continuation of rehabilitation training after hospital discharge has a positive impact on cognitive recovery.

A limitation of the present study was the relatively small sample size. There is a need for further clinical trials with larger sample sizes to confirm the results of this study, which may have implications for future clinical practice guidelines for JE treatment. Since this study is a retrospective study, the results of biochemical and serological tests are lacking to strengthen the claims. More data for MoCAs and DTI are also needed. In conclusion, the results of our study suggest that TAGMIC is an effective treatment for JE. Antiviral agents, glucocorticoids, and immunoglobulins act on different aspects of JE pathogenesis, and also have a synergistic effect. Moreover, an early and adequate course of therapy is essential for treatment success. Besides, the specific mechanisms of the drugs need to be further investigated.

## Data Availability Statement

The raw data supporting the conclusions of this article will be made available by the authors, without undue reservation.

## Ethics Statement

The studies involving human participants were reviewed and approved by Ethics Committee of the First Affiliated Hospital of Zhengzhou University (approval no. 2020-KY-077). The patients/participants provided their written informed consent to participate in this study. Written informed consent was obtained from the individual(s) for the publication of any potentially identifiable images or data included in this article.

## Author Contributions

WM and JT contributed to conception and design of the study. WM, JG, SZ, NS, XS, FL, WL, and JX organized the database. WM and JG performed the statistical analysis and wrote the first draft of the manuscript. All authors contributed to manuscript revision, read, and approved the submitted version.

## Funding

Joint construction project of Henan Medical Science and technology research plan (LHGJ20190087).

## Conflict of Interest

The authors declare that the research was conducted in the absence of any commercial or financial relationships that could be construed as a potential conflict of interest.

## Publisher's Note

All claims expressed in this article are solely those of the authors and do not necessarily represent those of their affiliated organizations, or those of the publisher, the editors and the reviewers. Any product that may be evaluated in this article, or claim that may be made by its manufacturer, is not guaranteed or endorsed by the publisher.
